# Soil-based environmental DNA enables detection of *Oncomelania hupensis quadrasi* and *Schistosoma japonicum* microhabitats for schistosomiasis japonica surveillance and control in the Philippines

**DOI:** 10.1186/s40249-025-01374-w

**Published:** 2025-10-30

**Authors:** Joseph E. Valencia, Marcello Otake Sato, Mark June Revolteado, Phoyphaylinh Prasayasith, Mario Jiz, Eleonor A. Cervantes, Ralph N. Aniceto, Marianette Inobaya, Pengfei Cai, Darren J. Gray, Catherine A. Gordon, Lydia R. Leonardo, Yasuhito Sako, Megumi Sato

**Affiliations:** 1 Graduate School of Health Sciences, Niigata University, Niigata, Japan; 2 Department of Health, Research Institute for Tropical Medicine, Muntinlupa, Philippines; 3 Division of Global Environment Parasitology, Faculty of Medical Technology, Niigata University of Pharmacy and Medical and Life Sciences, Niigata, Japan; 4 Molecular Parasitology Laboratory, QIMR Berghofer Medical Research Institute, Brisbane, Australia; 5 Faculty of Medicine, University of Queensland, Brisbane, Australia; 6 Institut Pasteur du Laos, Vientiane, Laos; 7 University of the Philippines Diliman, Quezon City, Philippines; 8 University of the East, Manila, Philippines; 9 University of the Philippines Manila, Manila, Philippines; 10 Division of Parasitology, Department of Infectious Diseases, Asahikawa Medical University, Asahikawa, Japan; 11 Centre for Tropical Health and Emerging Diseases, QIMR Berghofer Medical Research Institute, Brisbane, Australia; 12 School of Biomedical Sciences, The University of Queensland, Brisbane, Australia; 13 Population Health Program, QIMR Berghofer Medical Research Institute, Brisbane, Australia; 14 Applied Tropical and Molecular Parasitology Laboratory, QIMR Berghofer Medical Research Institute, Brisbane, Australia

**Keywords:** Environmental DNA, Soil-based eDNA, *Oncomelania hupensis quadrasi*, *Schistosoma japonicum*, Philippines, Ecohealth, Surveillance, Neglected tropical diseases

## Abstract

**Background:**

Schistosomiasis japonica, caused by *Schistosoma japonicum*, remains a significant public health concern in the Philippines, where 12.4 million people are at risk due to persistent transmission in endemic regions. The distribution of schistosomiasis is closely linked to the distribution of its snail intermediate host. This study aims to assess the potential of using soil samples to detect the environmental presence of *Oncomelania hupensis quadrasi* and *Schistosoma japonicum*.

**Methods:**

This cross-sectional observational study utilized a soil-based environmental DNA (eDNA) detection system for simultaneous detection of *O. h. quadrasi*, and *S. japonicum* mitochondrially encoded cytochrome c oxidase subunit 1 gene in multiplex quantitative real-time PCR and digital PCR platforms. A two-phase sample collection and testing were carried out in December 2023 (Phase 1) and March 2024 (Phase 2) across 30 selected sampling sites in Ekiran village, Leyte, Philippines. Wilcoxon two-sample/Mann-Whitney U test was used to determine whether there was a significant difference in eDNA presence and edaphic factors, while percent agreement was used to assess the concordance among methods.

**Results:**

This study reveals that *S. japonicum* eDNA can be detected in soil samples and confirms the strong applicability of soil-based eDNA for detecting *O. h. quadrasi* snails and even in sites without visible snail presence. Furthermore, it demonstrates the superiority of soil-eDNA system compared to classical malacological surveys in detecting *O. h. quadrasi* and *S. japonicum* microhabitats: in Phase 1, eDNA detected *O. h. quadrasi* and *S. japonicum* in 50% (3/6) and 66.67% (4/6) of sites, respectively, while malacological surveys detected them in only 50% and 16.67% (1/6) of sites. In Phase 2, eDNA detected *O. h. quadrasi* in 20% (6/30) of sites compared to only 10% (3/30) by malacological survey, and *S. japonicum* was detected only by eDNA in 10% (3/30) of sites. Among the measured soil parameters, only pH showed a statistically significant difference between eDNA-positive and eDNA-negative sites (*P* = 0.04).

**Conclusions:**

Soil-based eDNA sensitively detected *O. h. quadrasi* and *S. japonicum*, enabling scalable, non-invasive transmission site identification and outperforming traditional surveys without visible snails. Its ability to detect *S. japonicum* highlights its value for comprehensive schistosomiasis monitoring.

**Graphical Abstract:**

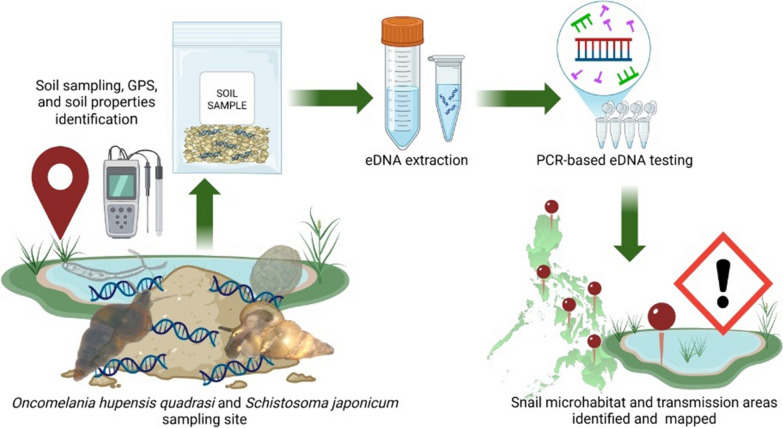

**Supplementary Information:**

The online version contains supplementary material available at 10.1186/s40249-025-01374-w.

## Background

Schistosomiasis japonica is a zoonotic neglected tropical disease endemic to Asia, including China, Indonesia, and the Philippines [1–3]. The disease affects 12.4 million people in the Philippines [4, 5], with the national prevalence of 4.68% as of 2019 based on focal survey using Kato-Katz [5]. The disease is endemic in three major islands of the country, Luzon, Visayas, and Mindanao [6, 7] with focal distribution in 12 regions, 28 provinces, and 196 towns [8]. An obligate snail-borne parasite *Schistosoma japonicum,* is the causative agent of schistosomiasis in the Philippines. Additionally, the freshwater snail *Oncomelania hupensis quadrasi* serves as its intermediate host [4, 9,10]. The snail is amphibious and persists in areas such as wet soil surface, rice fields, banks of streams, creeks, huge water-logged areas, and in pockets of medium and large rivers [11], notably in areas with perpetual wetness [12]. Furthermore, adult *O. h. quadrasi* spends most of its life in moist inland, however it stays mostly in water during breeding and egg laying [4].

A patent *S. japonicum-*infected *O. h. quadrasi* snail harbors the schistosome infective stage, cercaria. Thus, *O. h. quadrasi* persistence in the environment poses a significant public health risk in endemic communities [13]. Chemotherapy-based approach or mass drug administration (MDA) is still the main strategy to combat schistosomiasis in the Philippines. However, this strategy has been found to be inadequate to eliminate the disease [4, 14, 15]. As a response, the Philippine Department of Health, Schistosomiasis Control and Elimination Program (2018) adopted an integrated approach including MDA, innovative and intensified disease management, veterinary public health services, vector ecology and management, provision of safe water sanitation and hygiene, snail control, environmental sanitation, and health education [16, 17]. The current schistosomiasis environmental monitoring is solely conducted through classical malacological method, which is labor intensive and highly technical. Moreover, manual malacological method may easily fail to detect the snail presence given a focal and clumped distribution pattern of *O. h. quadrasi* [18], leading to inaccurate identification of probable transmission areas.

Environmental DNA (eDNA) detection systems have been applied to monitor the presence of organisms in the environment with promising results. The method was based on detecting the target organism’s DNA traces in the environment indicating its presence [19–21]. Earlier eDNA applications in parasitology mainly utilized water samples in detecting the environmental presence of parasites and vectors. These studies include the detection of *Opisthorchis viverrini* in Savannakhet, Lao PDR [22], *Schistosoma mansoni* in Madagascar [23], *S. japonicum* and *O. h. quadrasi* in the Philippines [24, 25]. Notably, one study in the Philippines utilized soil samples to detect *O. h. quadrasi* [26]. These earlier studies that applied eDNA technology to parasitology have demonstrated its potential, particularly in the aspect of eco-epidemiology. Building on the success of our previous eDNA studies, we applied a soil-based eDNA detection system to simultaneously detect the presence of *O. h. quadrasi* and *S. japonicum* at a community level. Soil samples were utilized in this study considering several factors such as *O. h. quadrasi* snail preferred habitat and its amphibious nature [11, 24], prolonged eDNA persistence in soil [27], and better preservation of eDNA in soil [28], which enable a sensitive detection of the target species presence in the environment.

This study aims to determine the *O. h. quadrasi* distribution and detect *S. japonicum* presence in the environment with high sensitivity using a soil-based eDNA system. Additionally, it seeks to provide an accurate *O. h. quadrasi* microhabitat map, and schistosomiasis risk areas at a community level. Overall, this will contribute to a better understanding of the disease dynamics within the community, which is crucial for program planning and policy making. By utilizing this approach, resources can be strategically focused on areas where they are significantly needed, thereby maximizing the impact of control efforts.

## Methods

### Study design and setting

**Fig. 1:**
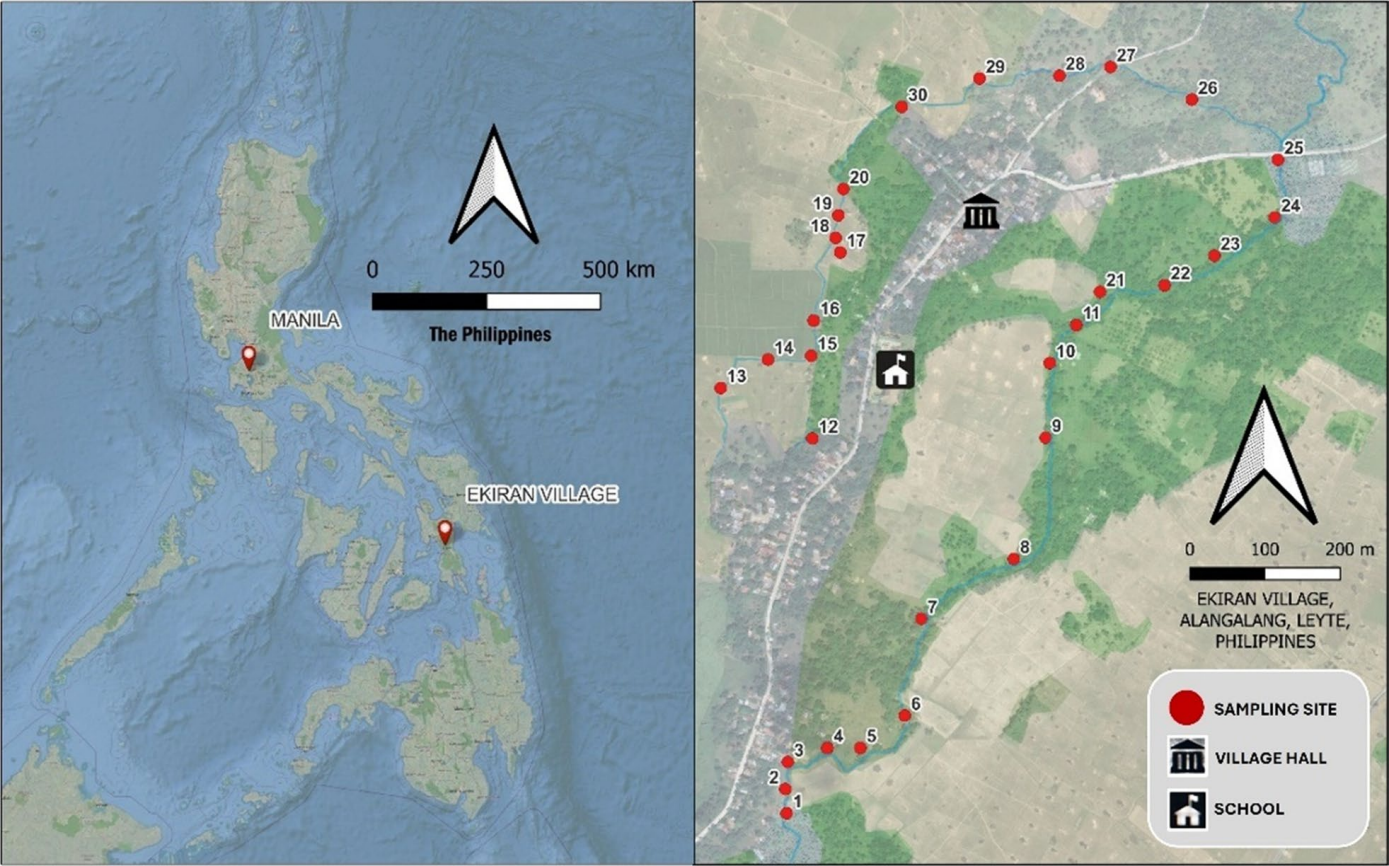
Geographical location of study site. The left map shows the Philippines with the location of Ekiran Village, while the right map highlights Ekiran Village, displaying the main waterways and the 30 soil eDNA sampling sites

This study utilized a cross-sectional observational study design to detect the presence of *S. japonicum* and *O. h. quadrasi* eDNA in soil samples from 30 selected sites in Ekiran village, Leyte, Philippines (Fig. 1)—one of the schistosomiasis endemic areas in the Philippines. Ekiran is a farming village surrounded by rice fields supplied by two main watercourses a natural stream situated in the eastern part of the village, providing a whole year-round water supply, and an irrigation system situated in the western part of the village. On the latter, water flow can be altered based on farming needs and may also be dry for some months. Small bamboo bridges and a low-angled soil slope with animals and human tracks were observed within the banks of the village’s watercourses, indicating access points for both humans and animals. The sample collection was carried out in December 2023 (Phase 1) and March 2024 (Phase 2).

### Soil sample collection

The samples were collected along the banks of the village watercourses using a systematic sampling approach to determine the sampling sites which were spaced approximately 20–100 m apart. Site selection was influenced by accessibility, particularly in areas with dense vegetation. The sampling was conducted in two phases: Phase 1 (P1) focused on six representative sites, while Phase 2 (P2) covered all 30 sites (Fig. 1). Phase 1 aimed to optimize the soil sampling distance and assess the extent of *O. h. quadrasi* and *S. japonicum* eDNA presence relative to their proximity to the watercourse bank. GPS coordinates of each site were recorded using Garmin GPS 64 Series device (Garmin Ltd., Kansas, USA).

Efforts were made to distribute sampling across a variety of microhabitats (Supplemental Table 1) within the study area; however, site selection was constrained by accessibility, particularly in areas with dense vegetation. Despite these limitations, the sampling design covered the entire village. Although this approach may have introduced some spatial sampling bias, standardized protocols for soil collection and DNA extraction were consistently applied across all sites to minimize detection variability and ensure data comparability.

Phase 1 sampling sites were selected based on historical malacological survey results, with three out of six sites (P1, P10, P21) having been previously identified as known snail habitats [25].

**Fig. 2:**
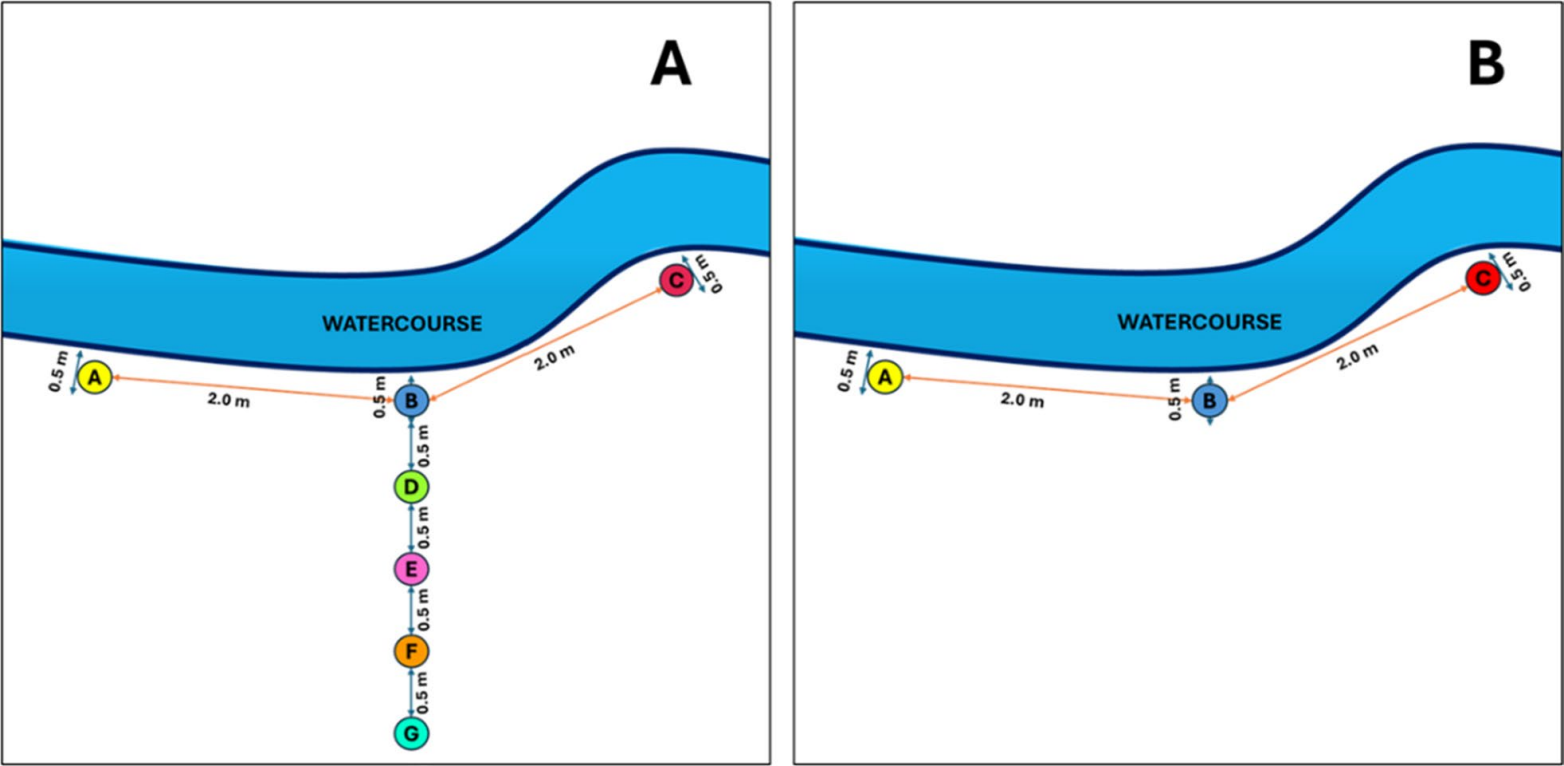
Cartographical representation of the sampling point placement relative to the watercourse bank. **A** Phase 1 (optimization of sampling distances), showing sampling points (SPs) A, B, and C within 0.5 m of the bank, and sampling points D (1 m), E (1.5 m), F (2 m), and G (2.5 m) from the watercourse bank. **B** Phase 2 (systematic sampling approach covering all the study area), with sampling points A, B, and C within 0.5 m of the watercourse bank, spaced 2 m apart

Soil sampling was conducted using two transect lines. The first transect was positioned parallel to the bank of the waterway, approximately within 0.5 m. Three sampling points were spaced two meters apart along this transect. Samples were collected from each sampling point and labeled as A, B, and C (Fig. 2A). The second transect line was positioned perpendicular to the midpoint of the first transect (sampling point-B). Along this second transect, samples were collected from four sampling points, with 0.5-m intervals, moving outward from the watercourse bank, and labeled as D, E, F, and G (Fig. 2A). The sampling distances were based on the observed moist areas along the watercourse bank, a crucial condition for snail survival [12]. Additionally, snail crawling activity observed in a previous study ranged from 0.425 to 0.562 cm per 10 min (or 0.612–0.809 m per day), depending on the time of day [10].

At some sites adjacent to rice fields, only three samples (A, B, and C) were collected, as outward soil sampling was not feasible due to accessibility constraints and the potential risk of damaging the rice crop. Approximately 100 g of soil was collected per sampling point in each sampling site. The soil samples were then kept in a properly labelled resealable plastic bag and kept in a Styrofoam box with ice to maintain a low temperature during transport to the laboratory for processing. In Phase 2, thirty sampling sites were selected (Fig. 1) including the six sampling sites from Phase 1. Distances from each sampling site are approximately 20–100 m. Based on the Phase 1 results, the number of sampling points in Phase 2 was reduced to three, excluding the sampling points in the second transect. The sampling points were spaced two meters apart (Fig. 2B).

### Malacological survey and *S. japonicum* snail infection determination

The malacological survey was conducted using a time-based sampling method [12, 25]. The collected snails were kept in a properly labelled resealable plastic bag with moist paper towel. Samples were kept in a Styrofoam box with ice for transport to the laboratory. They were then morphologically examined and individually crushed into a few drops of distilled water. The crushed *O. h. quadrasi* snails were then examined for the presence of *S. japonicum* cercariae under the microscope. Snails containing a characteristic furcocercous cercariae were considered infected with *S. japonicum* [9].

### Soil moisture, pH, and temperature determination

Soil moisture was determined by the gravimetric method. A 10 g sample aliquot of soil was dried in an oven at 60 °C for 48 h. The low temperature setting was selected due to the high organic content of the soil. Percent moisture was then calculated by taking the difference of initial and dried weight divided by the initial weight, and the results were expressed as percent moisture [29].

Soil pH and temperature were determined and recorded onsite using a soil meter monitor (Shinwa Digital Soil Acidity Meter 72730®, Shinwa Rules Co., Ltd., Niigata, Japan). The device was operated according to the manufacturer’s instructions and placed at the representative sampling point (SP-B).

### eDNA extraction

Soil eDNA extraction was performed following the method described by Sakata et al. [30]. The method includes an initial alkaline extraction and ethanol precipitation, followed by using DNeasy PowerSoil Pro Kits® (QIAGEN, Hilden, Germany) in accordance with the manufacturer’s instructions. The eDNA extracts were then kept at −30 °C until testing.

### Primers and probes

The primers and probes were adapted from the validated method of Revolteado et al*.* [25]. A premixed set (Integrated DNA Technologies, Inc., Coralville, Iowa, USA) targeting the mitochondrially encoded cytochrome c oxidase subunit 1 (MT-CO1) gene was used for both *O. h. quadrasi* and *S. japonicum*. The sequences for *O. h. quadrasi* were: Forward – 5’-GCATGTGAGCGGGGCTAGTAGGC-3’; Reverse – 5’-AAGCGGAACCAATCAGTTGCC-3’; and Probe – 5'-/56-FAM/AGGACTGAC/ZEN/CTAACTCTGCAC/31ABkFQ/-3' (amplicon size: 187 bp). For *S. japonicum*, the sequences were: Forward – 5’-TTTGATAACTAATCACGGTATAGCAA-3’; Reverse – 5’-CGAGGCAAAGCTAAATCACTC-3’; and Probe – 5'-/5HEX/TTTTGGTAA/ZEN/ATATCTTCTTCCG/31ABkFQ/-3' (amplicon size: 119 bp).

### Quantitative real-time polymerase chain reaction (qPCR) assay

The qPCR was carried out in a 30 µl reaction volume as previously described [25]. Briefly, each reaction was composed of 1 × *Taq*Man® Environmental Master Mix 2.0 (Applied Biosystems, California, USA), 333 nmol/L (primer) and 167 nmol/L (probe) of each MT-CO1 (*O. h. quadrasi, S. japonicum*) primer–probe mix (Integrated DNA Technologies, Inc., Coralville, Iowa, USA), and 5 µl of DNA template. Cycling conditions were set as follows: initial denaturation at 95 °C for 10 min, 50 cycles of two-step PCR at 95 °C for 15 s, and 60 °C for 60 s. The assay was performed using a Magnetic Induction Cycler (MIC, Bio Molecular Systems, Upper Coomera, Queensland, Australia), a portable thermocycler fit for field applications. Samples were tested three times with both positive and negative controls included in each run. Amplifications above threshold for eDNA samples were considered positive, otherwise negative.

### Digital PCR (dPCR) assay

dPCR was performed using QIAcuity One® (QIAGEN, Hilden, Germany). The reactions were done on a QIAcuity Nanoplate 26 k 24-well plate. The assay was run following the manufacturer’s recommendations using a 40 µl reaction consisting of 1 × Probe PCR Master Mix (QIAGEN, Hilden, Germany), 500 nmol/L (primer) and 250 nmol/L (probe) of each MT-CO1 (*O. h. quadrasi, S. japonicum*) primer-probe mix (Integrated DNA Technologies, Inc., Coralville, Iowa, USA), and 5 µl of DNA template. The cycle temperature was set based on the manufacturer’s recommendations at initial denaturation of 95 °C for 2 min, 50 cycles of two-step PCR at 95 °C for 15 s, and 60 °C for 60 s. Digital PCR positive in one or more partitions was considered positive, otherwise negative.

### Statistical analysis and mapping

Field and laboratory data were encoded in Microsoft Excel (Version 2504 Build 16.0.18730.20186; Microsoft corporation, Redmond, USA) for data management and preliminary processing including the percent agreement. The Cq values and target DNA copy numbers per microliter from qPCR and dPCR were reported as a mean of positive results from triplicate samples. Continuous, non-normally distributed soil parameters were presented as median (interquartile range). The *P* value of eDNA positivity and edaphic factors were calculated using the Wilcoxon two-sample/Mann-Whitney U test on JMP®, V 18 (SAS Institute, North Carolina, USA). The percentage agreement (concordance) of the results of two methods, malacological survey and eDNA, was also determined. The eDNA results were mapped using QGIS V3․32․3 Lima (QGIS Association, Zurich, Switzerland). Statistical significance was set at *P* < 0.05.

## Results

### Soil-based eDNA detection using qPCR and dPCR

Both qPCR and dPCR were successful in detecting *O. h. quadrasi* and *S. japonicum* eDNA from soil samples. The eDNA positivity rate was higher when using qPCR compared to dPCR in both study phases (Tables 1 and 2). A qPCR-positive sample with a Cq of more than 37.02 becomes negative when tested using dPCR. The lowest detection in dPCR was recorded at 1.01 copies of target DNA per microliter, which corresponds to 2/26,000 positive partitions in the Nanoplate well (Table 2).

**Table 1:** Phase 1 (optimization of sampling distances) eDNA and malacological survey positivity and concordance

Site ID	Malacological survey		eDNA method			
	*Ohq* observed (*n*)	*Sj-*infected *Ohq* (*n*)	*Ohq* qPCR Cq (x̄)	*Sj* qPCR Cq (x̄)	*Ohq* dPCR (copies/μl) (x̄)	*Sj* dPCR (copies/μl) (x̄)
01	10	1 (10%)	36.40	38.90	3.41	–
10	5	–	–	31.52	–	31.02
16	–	–	39.07	–	–	–
21	10	–	38.42	–	–	–
25	–	–	–	38.10	–	–
28	–	–	–	40.43	–	–
Agreement	NA	NA	66.67% (4/6)	50% (3/6)	66.67% (4/6)	66.67% (4/6)
Positivity rate	50% (3/6)	16.67% (1/6)	50% (3/6)	66.67% (4/6)	16.67% (1/6)	16.67% (1/6)

The x̄ values (Cq, copies/μl) represent the arithmetic mean of three replicate measurements per positive site

The table summarizes the Phase 1 positivity rates and percent agreement (concordance between) eDNA detection and malacological survey results for *O. h. quadrasi* (*Ohq*) and *S. japonicum* (*Sj*). The comparison of eDNA detection and classical malacological survey results is also showed, highlighting the higher *S. japonicum* eDNA detection rates of the eDNA method. Abbreviates: —means no detection, *Cq* cycle quantification.

**Table 2:** Phase 2 eDNA and malacological survey positivity and concordance

Site ID	Malacological survey		eDNA method			
	*Ohq* observed (*n*)	*Sj-*infected *Ohq* (*n*)	*Ohq* qPCR Cq (x̄)	*Sj* qPCR Cq (x̄)	*Ohq* dPCR (copies/μl) (x̄)	*Sj* dPCR (copies/μl) (x̄)
01	3	–	31.99	–	20.52	–
02	–	–	–	–	–	–
03	–	–	–	–	–	–
04	–	–	–	37.95	–	–
05	–	–	39.23	–	–	–
06	–	–	–	–	–	–
07	–	–	–	–	–	–
08	–	–	–	–	–	–
09	–	–	–	–	–	–
10	13	–	37.02	–	1.01	–
11	–	–	40.32	–	–	–
12	–	–	–	–	–	–
13	–	–	–	–	–	–
14	–	–	–	–	–	–
15	–	–	–	–	–	–
16	–	–	–	–	–	–
17	–	–	–	–	–	–
18	–	–	–	–	–	–
19	–	–	37.66	–	–	–
20	–	–	–	–	–	–
21	1	–	38.17	–	–	–
22	–	–	–	–	–	–
23	–	–	–	–	–	–
24	–	–	–	43.38	–	–
25	–	–	–	–	–	–
26	–	–	–	–	–	–
27	–	–	–	37.68	–	–
28	–	–	–	–	–	–
29	–	–	–	–	–	–
30	–	–	–	–	–	–
Agreement	NA	NA	90.00% (27/30)	90.00% (27/30)	96.67% (29/30)	100% (30/30)
Positivity rate	10.00% (3/30)	0 (0/30)	20.00% (6/30)	10.00% (3/30)	6.67% (2/30)	0 (0/30)

The x̄ values (Cq, copies/μl) represent the arithmetic mean of three replicate measurements per positive site

The table shows positivity rates and percent agreement (concordance) between Phase 2 eDNA detection and malacological survey results for *O. h. quadrasi* (*Ohq*) and *S. japonicum* (*Sj*). The table also compares eDNA detection with classical malacological survey results, emphasizing the superior detection rates of the eDNA method. Abbreviations: —no detection, *NA* not applicable, *Cq* cycle quantification.

### Soil sampling distance and eDNA detectability

In Phase 1, eDNA of either *O. h. quadrasi* or *S. japonicum* was detected within 0.5–2.5 m of the watercourses’ bank, with no significant difference between proximity and eDNA positivity (*n* = 20, *P* > 0.05) (Supplemental Fig. 1). The result from Phase 1 showed that *S. japonicum* eDNA was detected in four out of six reference sampling sites (66.67%) while *O. h. quadrasi* eDNA was detected in three out of six reference sampling sites (50%). The agreement for malacological survey and combined eDNA method was 66.67% (4/6) and 50% (3/6), for *O. h. quadrasi* and *S. japonicum* detection, respectively. The qPCR Cq ranges from 36.40 to 39.07 for *O. h. quadrasi* and 38.10–40.43 for *S. japonicum.* Furthermore, dPCR result showed detection of 3.41 and 31.02 copies/µL of *O. h. quadrasi* and *S. japonicum* eDNA, respectively (Table 1).

### *O. h. quadrasi* and *S. japonicum* environmental DNA presence

In phase 2, 30 sampling sites were included, covering the entirety of the village following the potential snail microhabitats along the village’s watercourses. Using the soil-based eDNA detection system, *O. h. quadrasi* eDNA was detected in six out of 30 sampling sites (20%), while *S. japonicum* eDNA was detected in three out of 30 sampling sites (10%). The agreement between malacological survey and combined eDNA method was 90% (27/30) for both *O. h. quadrasi* and *S. japonicum* detection. Fifty percent (3/6) of *O. h. quadrasi* eDNA positive sampling siteshas no presence of snails based on malacological survey. eDNA positivity for both target species was found in seven out of nine sites (77.77%) mostly near the village’s natural stream. The qPCR Cq for *O. h. quadrasi* ranges from 31.99 to 40.32 and *S. japonicum* ranges from 37.68 to 43.38, respectively. Positive dPCR of *O. h. quadrasi* ranges from 1.01 to 20.52 eDNA copies/ul, no *S. japonicum* eDNA were detected by dPCR in Phase 2. Notably, all surveyed sites containing snails tested positive using the eDNA detection system, even in areas where only single snails were found in Phase 2.

### Edaphic factors

The result of the edaphic factors determinations are as follows: pH 5.25 (IQR: 5.00 − 6.21), percent moisture 40 (IQR: 40 − 50), and temperature (℃) 26. (IQR: 25 − 27). Among these factors, only pH showed a significant difference (*P* = 0.04) between eDNA positive and negative sites (Supplemental Fig. 2). The pH of the soil across the sites examined ranges from 4.50 to 7.40. The *O. h. quadrasi* and *S. japonicum* eDNA positives were observed in soils with pH between 5.00 and 7.40 with the majority (63%) falling within pH 5.00–6.00. The median pH for eDNA-positive and negative sites is 5.50 (IQR: 5.00 − 6.40), and 5.00 (IQR: 5.00 − 5.50), respectively.

### Distribution of *O. h. quadrasi* microhabitats and *S. japonicum* presence as detected by soil-based eDNA system

**Fig. 3:**
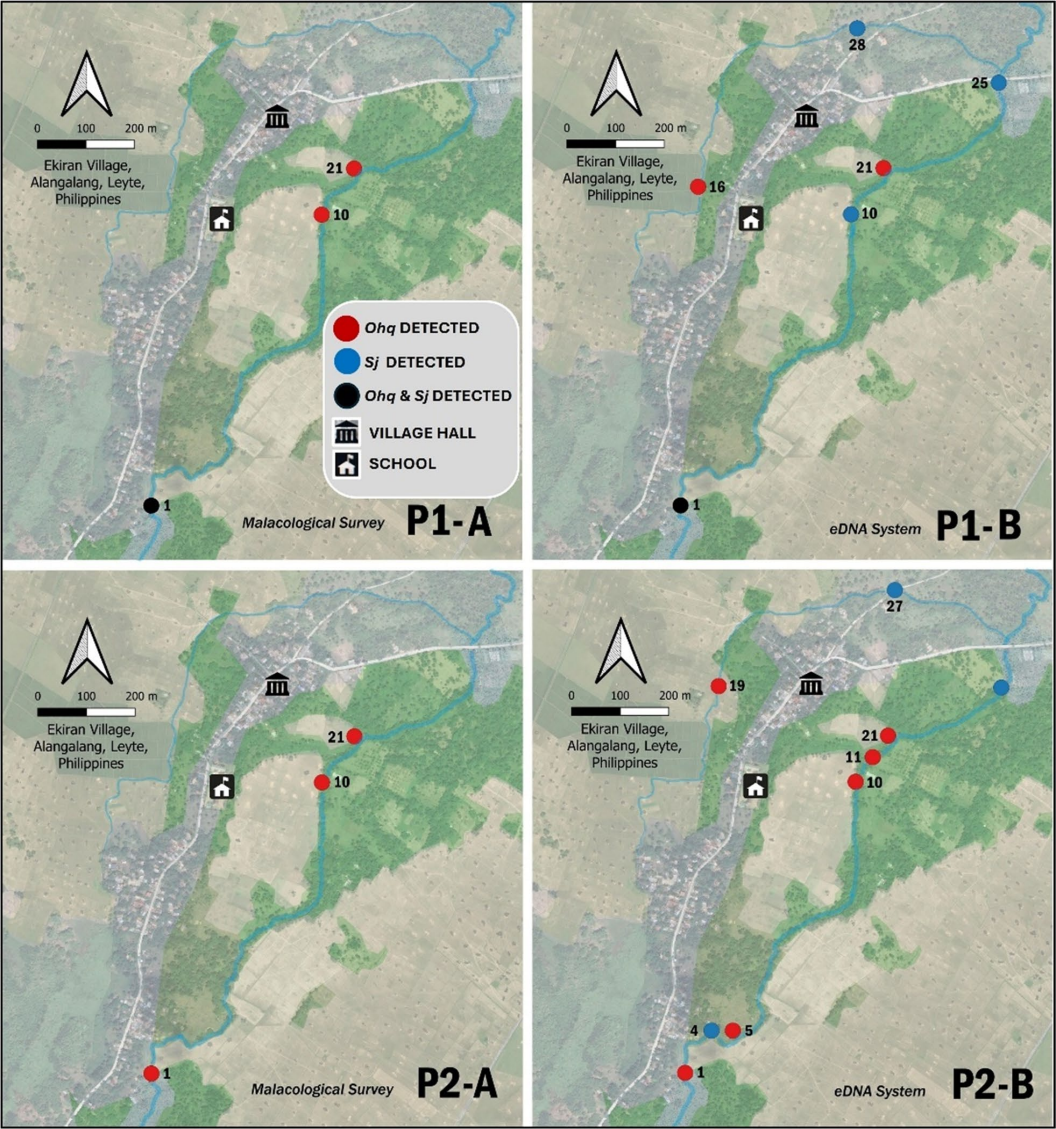
Map showing *O. h. quadrasi* microhabitats and *S. japonicum* presence in P1 and P2 survey at Ekiran Village. P1-A & P2-A – map of malacological survey results; P1-B and P2-B – map of eDNA detection system results. Red dots indicate *O. h. quadrasi* detected, blue dot represents sites where *S. japonicum* was detected, and Black dot indicates both *O. h. quadrasi* and *S. japonicum* were detected. These maps provide a visual comparison of the two survey methods, highlighting the increased detection sensitivity of the eDNA system, especially in areas where traditional malacological surveys failed to detect *S. japonicum*. The spatial distribution of *O. h. quadrasi* and *S. japonicum* across both phases emphasizes the utility of soil-based eDNA in identifying microhabitats and transmission zones, particularly in areas with low snail populations or where snails were not directly observed

*O. h. quadrasi* and *S. japonicum* were mostly detected in sites adjacent to the village’s watercourse with perpetual water supply. The distribution of *O. h. quadrasi* microhabitats exhibited a random and clumped distribution. Combined (Phase 1 and 2) detection rates for *O. h. quadrasi* and *S. japonicum* were 10% (3/30) to 50% (4/6) in malacological survey, while detection rate ranged from 30% (9/30) to 100% (6/6) in soil-based eDNA method (Fig. 3).

## Discussion

This study demonstrated the application of soil-based eDNA in detecting *O. h. quadrasi* and *S. japonicum* from samples collected across several proximity ranges from watercourses in Ekiran Village. These findings have provided key insights into eDNA detectability and its correlation with the presence of snails and parasites in the environment. The sensitive identification of *O. h. quadrasi* microhabitats and *S. japonicum* presence using soil-based eDNA identifies schistosomiasis transmission risk areas – a crucial information to mitigate human and animal exposure. This system can also be applied to monitor schistosomiasis in areas with zero transmission, regions nearing elimination, and potential new endemic areas. Furthermore, the soil-based eDNA system was able to detect *O. h. quadrasi* and *S. japonicum* eDNA from soil in watercourses banks up to 2.5 m, with no significant difference between detection and proximity. This suggests the stability of *O. h. quadrasi* and *S. japonicum* eDNA within this range which is likely influenced by soil retention properties [27, 28]. The eDNA detectability of *O. h. quadrasi* and *S. japonicum* in Phase 1 was 50% (3/6) and 66.67% (4/6) respectively, suggesting the persistence of these target organisms in environmentally conducive sites. The agreement between malacological survey and soil-based eDNA detection is 66.67% (4/6) for *O. h. quadrasi* and 50% (3/6) for *S. japonicum* indicating that eDNA can complement or even surpass the traditional method, capturing the organism’s presence that could possibly be overlooked by direct observations.

The optimized sampling method was applied in Phase 2, during which thirty sites were sampled, covering the areas adjacent to the village’s main waterways. The eDNA detection rates for *O. h. quadrasi* and *S. japonicum* were 20% (6/30) and 10% (3/30), respectively. These detections were lower than those in Phase 1 likely reflecting broader physical environmental variations and dilution effects of increasing sampling sites. However, there is a high (90%, 27/30) agreement between the malacological survey and eDNA detection in Phase 2 emphasizing the reliability of eDNA as a surveillance tool. Notably, 50% (3/6) of *O. h. quadrasi* and 100% (3/3) of *S. japonicum* eDNA-positive sites in Phase 2 were not identified by malacological surveys, validating the ability of soil-based eDNA system to detect traces of snail and parasite presence in areas where live specimens were not directly observed. These findings are particularly valuable in schistosomiasis surveillance, as detecting *O. h. quadrasi* and *S. japonicum* presence in the environment can facilitate early and specific mitigation efforts.

The usefulness of the novel approach of testing eDNA using dPCR was also demonstrated in this study (Tables 1, 2). A successful detection and quantification of DNA from the environmental samples shows its potential in future eco-epidemiological applications. This method has detected the presence of eDNA in as low as one copy per μl. In spite of its lower detection rate compared with qPCR, the dPCR advantage relies on its capacity to separate reactions into thousands of partitions enabling absolute quantifications and enhanced detection of an inhibitor-rich sample. These initial results will provide a foundation for future studies and applications of dPCR in eDNA detection systems.

In the analysis of edaphic factors, pH was found to have a significant difference (*P* < 0.05) between sites with (Median = 5.50, IQR: 5.00 − 6.40) and without (Median = 5.00, IQR: 5.00 − 5.50) eDNA detection. Water-based eDNA studies have found that lower water pH (pH 4) negatively influenced eDNA detection [31]. Conversely, a study on the effect of bacteria and pH to eDNA detectability in water showed an opposite result, showing eDNA decay rate proportional to the increase in pH [32]. In combination with other factors, such as ultraviolet light and nucleases, pH has a more direct and measurable effect on eDNA degradation in water compared to soil. While pH influences eDNA degradation in both environments, the aquatic environment’s homogenous composition and fewer buffering agents allow for clearer and more rapid pH-related impacts on eDNA persistence than in soil [33–35]. These factors, alongside pH, influence the eDNA degradation and persistence in soil samples, which may affect detection efficiency. Furthermore, *O. h. quadrasi* and *S. japonicum* require a specific pH to persist. An optimal pH of 7.6 was found to be favorable for cercarial shedding while a higher pH (> 9) and lower pH (< 4.6) have shown to reduce *O. h. quadrasi* productivity [4, 12]. Soil moisture and temperature did not show significant associations, possibly because of the relatively stable environmental conditions at the study sites.

The distribution pattern of *O. h. quadrasi* and *S. japonicum* eDNA was primarily clumped and random, with detections concentrated in areas near watercourses with a continuous water supply. This aligns with the known habitat preferences of *O. h. quadrasi*, which thrives in moist environments with stable water availability [12]. The overall detection rates varied depending on the method used. The traditional malacological survey identified *O. h. quadrasi* in 10% (3/30) and *S. japonicum* presence in 3.33% (1/30) of the total sampling sites, while soil-based eDNA method yielded higher detection rates at 23.33% (7/30), and 20% (6/30) for *O. h. quadrasi* and *S. japonicum*, respectively. The ability of soil-based eDNA to detect *O. h. quadrasi*, even in areas with only a single snail were observed, further reinforces its potential as a sensitive and non-invasive monitoring tool.

Our study highlights the advantages of soil-based eDNA detection system in detecting *O. h. quadrasi* snails. The amphibious habits of snails in which adults spend most of their time in moist soil [4, 12, 26] allow us to capture their actual presence in the environment. In addition, the better preservation of eDNA in soil [27, 28] extends the detection window, thereby increasing detection sensitivity leading to more robust epidemiological data and risk assessment.

The laboratory observations of the laboratory-bred *O. h. quadrasi* show diphasic growth. A highly aquatic stage during the hatchling period, and the highly amphibious stage from early adulthood to the rest of their adult life. This stage-dependent habitat preference increases the possible detectability of the target organism in moist soil. Moreover, the youngest *S. japonicum*-infected *O. h. quadrasi* observed in the field measured 3.0 mm [4]. This is consistent with an early adult size of laboratory-bred snails. Given the biological behavior of snails, infected young snails are more likely to inhabit moist inland areas.This provides the biological rationale for using soil-based eDNA detection as a suitable method for detecting the environmental presence of *O. h. quadrasi* and *S. japonicum*. However, adult snails also move to water during egg-laying, which peaks during rainfall and flooding (rainy season), as well as during the breeding period which peaks during the month of May and decreases from June to July but continues up to October [4]. These events may increase the probability of detecting the target organisms’ eDNA in water thereby suggesting an alternative approach of utilizing water samples during these periods [25].

To address potential spatial sampling bias, several measures were taken to minimize variability and ensure reliable detection. As part of the optimized sampling design, triplicate soil samples were collected at each site within a standardized range of 0.5 m from the waterway bank. This was based on the known behavior and lateral movement of *O. h. quadrasi* from aquatic to moist terrestrial zones. The sampling design aligns with the snail’s natural habitat preference, particularly during its amphibious adult stage, when it tends to remain close to water bodies while seeking moist soil for shelter and oviposition [10, 12]. By targeting this ecologically relevant zone and applying uniform sampling distances, volumes, and collection techniques, the study aimed to reduce the risk of false negatives due to spatial variability. Furthermore, consistent DNA extraction and processing protocols were used across all sites to ensure comparability. Despite these efforts, some inherent variability due to microhabitat differences and patchy organism distributions may remain. To further optimize detection and address this limitation, future studies could explore stratified or adaptive sampling designs and integrate spatial modeling tools to enhance ecological representation and surveillance coverage.

The successful utilization of soil-based eDNA to detect *O. h. quadrasi* and *S. japonicum* in our study builds upon and strengthens previous findings that primarily utilized aquatic sampling matrices. In Madagascar, for example, *S. mansoni* eDNA was successfully detected from water samples collected at human-use sites using a MT-CO1 targeted qPCR assay, and its presence was confirmed by traditional malacological surveys through the visual collection of *Biomphalaria pfeifferi,* the intermediate host [23]. In the Philippines, subsequent applications of water-based eDNA detection using species-specific qPCR assays similarly identified *O. h. quadrasi* and *S. japonicum,* showing high concordance with malacological data while also revealing additional transmission foci that had gone undetected by conventional methods [24, 25].

Beyond schistosomes detection, eDNA-based surveillance has proven effective for detecting other trematode species and their snail intermediate hosts. For instance, *Opisthorchis viverrini* has been monitored in freshwater habitats using both qPCR and multi-marker eDNA assays, demonstrating the method’s adaptability across different parasite-host systems and environmental settings [22, 36]. In veterinary parasitology, studies have used eDNA to detect *Fasciola hepatica* and *Calicophoron daubneyi*, as well as their intermediate host *Galba truncatula*, in livestock-grazed wetland environments—even in the absence of observable snails [37]. These examples highlight the growing relevance of eDNA across trematode surveillance and control efforts.

While most of these studies relied on water as the sampling medium, our investigation is among the first to successfully apply soil-based eDNA for the detection of *S. japonicum*. This advancement not only extends the application of the soil-based approach initially introduced for *O. h. quadrasi* [26] but also broadens its utility to include the parasite itself. Importantly, the soil-based method offers a valuable alternative for terrestrial or amphibious snail habitats ecological settings where aquatic-based monitoring and visual detection methods often fall short due to seasonal variability, inaccessibility, or low population density.

Despite its advantages, the soil-based eDNA detection system also presents several limitations that must be addressed for broader application. The method is technically demanding, requiring laboratory-grade equipment, specialized reagents, and trained personnel. Strict contamination control procedures are essential throughout whole process, from field collection to DNA extraction and amplification which can be both technically and logistically challenging. The high sensitivity of PCR based assays comes at the cost of expensive consumables and instrumentation, limiting its immediate feasibility for routine surveillance in resource-constrained endemic areas. Nevertheless, the system remains cost-effective due to the rapid implementation of surveys, which saves time and manpower resources, especially since the field collection process does not require intensive labor. An additional challenge involves environmental inhibitors commonly present in soil, such as humic substances can reduce detection efficiency, making robust sample pre-treatment and inhibitor-resistant protocols critical. A further challenge is the inability of eDNA-based approaches to distinguish between viable organisms and remnant DNA, such as parasite DNA introduced via fecal contamination. This necessitates careful interpretation of positive detections, ideally in conjunction with contextual ecological data. To optimize the current system, future research should prioritize refining eDNA sampling protocols based on the biology and ecology of the target organisms. This includes selecting appropriate sample matrices (e.g., soil vs. water), determining optimal seasonal sampling windows, and improving DNA extraction and amplification workflows to maximize sensitivity while reducing costs. Continued investigation into environmental variables such as pH, temperature, and soil composition is also warranted, as these factors affect eDNA degradation and persistence.

Ultimately, integrating soil-based eDNA detection into existing schistosomiasis surveillance frameworks can expand spatial coverage and increase sensitivity for detecting parasite and snail presence. Compared to traditional malacological surveys, soil-based eDNA is more practical and sensitive, capable of identifying *O. h. quadrasi* even in low-density populations. By complementing conventional methods, it offers a valuable tool for identifying high-risk areas—especially in the aftermath of natural disasters such as typhoons and flooding, which may influence snail and parasite dispersal. This approach holds promise for guiding targeted interventions in both endemic and near-elimination settings.

## Conclusions

This study highlights the usability and effectiveness of soil-based eDNA system for simultaneously detecting *O. h. quadrasi* and *S. japonicum* in schistosomiasis endemic community in the Philippines. Soil-based eDNA was shown as a reliable and non-invasive tool for identifying schistosomiasis risk areas, and probable transmission sites, with high detection rates even in low-density snail populations and even in areas where snails were not observed through traditional malacological surveys. The successful application of this method points to its potential for improving surveillance in regions affected by environmental disruptions, such as flooding. In addition, this study reveals the importance of environmental factors, particularly pH, in influencing eDNA detectability, which warrants further investigation. The eDNA system offers a promising future tool for eco-epidemiological applications, particularly in areas nearing transmission interruption or facing new endemic risks. Soil-based eDNA offers a more sensitive, cost-effective, and scalable approach for schistosomiasis monitoring compared to classical malacological surveys. Moreover, soil-based eDNA applications can guide future strategic approaches, allowing targeted intervention to reduce human and animal exposure in endemic communities. Future research should focus on refining detection protocols, correlating biological behaviors with environmental sampling, and understanding the environmental factors affecting eDNA persistence.

## Supplementary Information

Supplementary material 1. Supplemental Figure 1: Comparison between sampling proximity and overall eDNA positivity in phase 1 using Wilcoxon two-sample/Mann-Whitney U test.

Supplementary material 2. Supplemental Figure 2: Comparison of edaphic factors between eDNA-positive and eDNA-negative sites using Wilcoxon two-sample/Mann-Whitney U test.

Supplementary material 3. Supplemental Table 1: Characteristics of the 30 soil sampling sites based on ocular observations in Ekiran Village, Alangalang, Leyte, Philippines.

## Data Availability

The data supporting the findings of this study is available upon request to the corresponding author. All data relevant to the study are included in the article or uploaded as supplementary information.

## Abbreviations

MT-CO1: Mitochondrially encoded cytochrome c oxidase subunit 1
dPCR: Digital polymerase chain reaction
eDNA: Environmental DNA (deoxyribonucleic acid)
*Ohq*: *Oncomelania hupensis quadrasi*
P1: Phase 1
P2: Phase 2
pH: Power of hydrogen
qPCR: Quantitative real-time polymerase chain reaction
*Sj*: *Schistosoma japonicum*
