# Supplementary material for: Soil-based environmental DNA enables detection of Oncomelania hupensis quadrasi and Schistosoma japonicum microhabitats for schistosomiasis japonica surveillance and control in the Philippines
Source: Infect Dis Poverty. 2025 Oct 30;14:110. doi: 10.1186/s40249-025-01374-w (PMC12574143; doi:10.1186/s40249-025-01374-w)
Supplement: Supplementary file 1 — Supplementary material 1. Supplemental Figure 1: Comparison between sampling proximity and overall eDNA positivity in phase 1 using Wilcoxon two-sample/Mann-Whitney U test. [file 40249_2025_1374_MOESM1_ESM.pptx]

## Slide 1
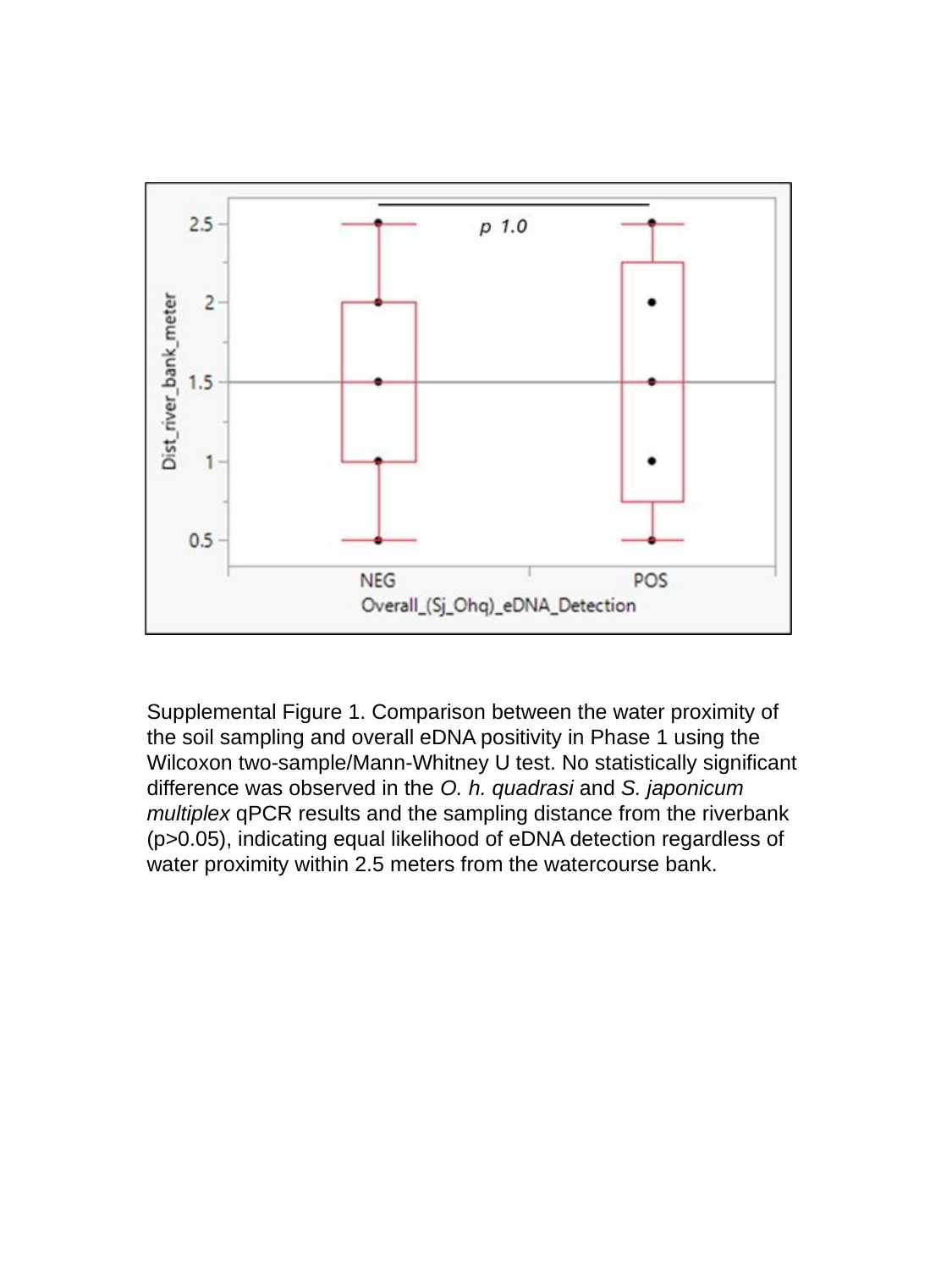

Supplemental Figure 1. Comparison between the water proximity of the soil sampling and overall eDNA positivity in Phase 1 using the Wilcoxon two-sample/Mann-Whitney U test. No statistically significant difference was observed in the O. h. quadrasi and S. japonicum multiplex qPCR results and the sampling distance from the riverbank (p>0.05), indicating equal likelihood of eDNA detection regardless of water proximity within 2.5 meters from the watercourse bank.
