# Supplementary material for: Soil-based environmental DNA enables detection of Oncomelania hupensis quadrasi and Schistosoma japonicum microhabitats for schistosomiasis japonica surveillance and control in the Philippines
Source: Infect Dis Poverty. 2025 Oct 30;14:110. doi: 10.1186/s40249-025-01374-w (PMC12574143; doi:10.1186/s40249-025-01374-w)
Supplement: Supplementary file 2 — Supplementary material 2. Supplemental Figure 2: Comparison of edaphic factors between eDNA-positive and eDNA-negative sites using Wilcoxon two-sample/Mann-Whitney U test. [file 40249_2025_1374_MOESM2_ESM.pptx]

## Slide 1
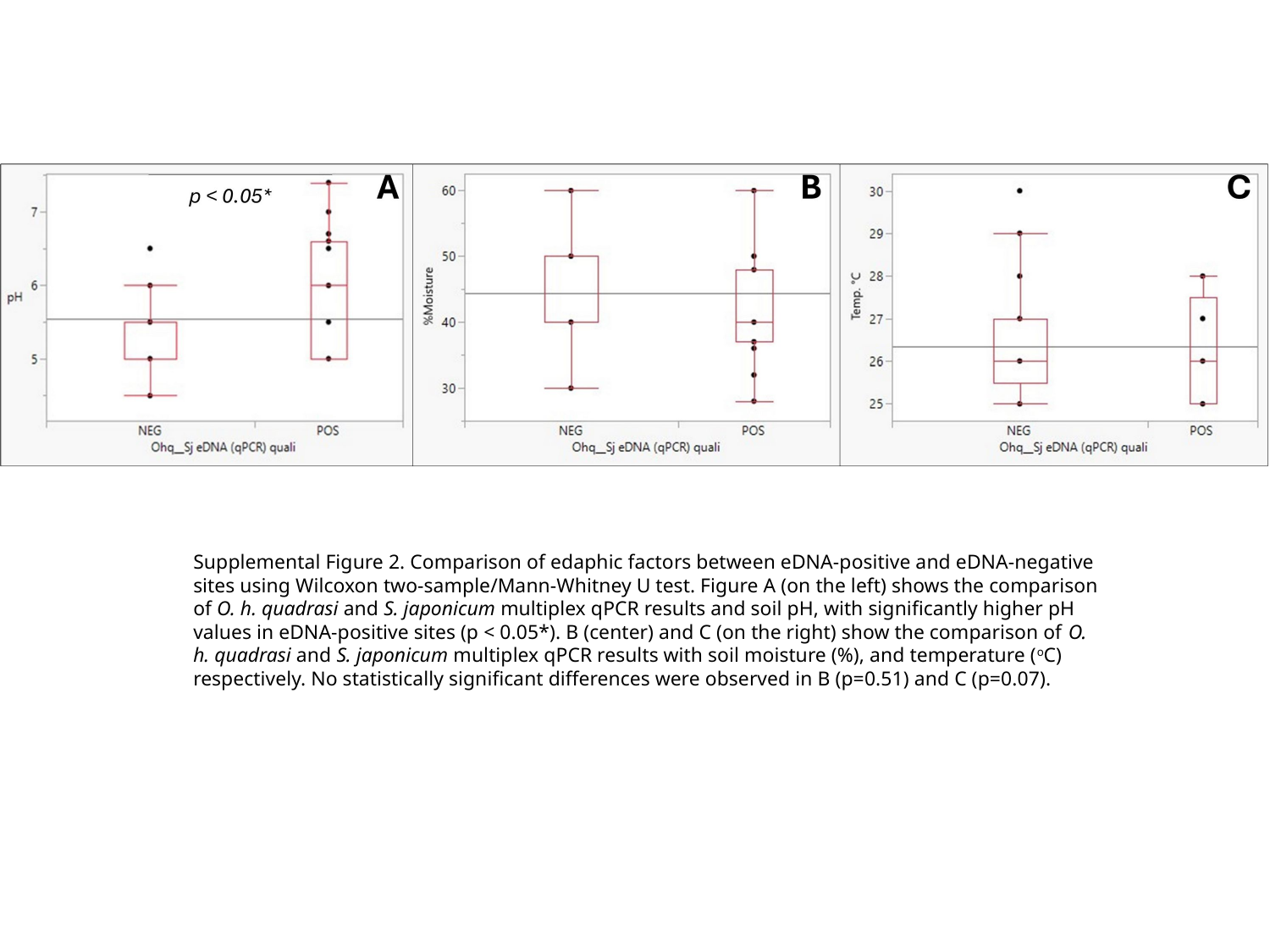

Supplemental Figure 2. Comparison of edaphic factors between eDNA-positive and eDNA-negative sites using Wilcoxon two-sample/Mann-Whitney U test. Figure A (on the left) shows the comparison of O. h. quadrasi and S. japonicum multiplex qPCR results and soil pH, with significantly higher pH values in eDNA-positive sites (p < 0.05*). B (center) and C (on the right) show the comparison of O. h. quadrasi and S. japonicum multiplex qPCR results with soil moisture (%), and temperature (oC) respectively. No statistically significant differences were observed in B (p=0.51) and C (p=0.07).
