# Supplementary material for: Soil-based environmental DNA enables detection of Oncomelania hupensis quadrasi and Schistosoma japonicum microhabitats for schistosomiasis japonica surveillance and control in the Philippines
Source: Infect Dis Poverty. 2025 Oct 30;14:110. doi: 10.1186/s40249-025-01374-w (PMC12574143; doi:10.1186/s40249-025-01374-w)
Supplement: Supplementary file 3 — Supplementary material 3. Supplemental Table 1: Characteristics of the 30 soil sampling sites based on ocular observations in Ekiran Village, Alangalang, Leyte, Philippines. [file 40249_2025_1374_MOESM3_ESM.docx]

| **SITE ID** | **General Collection Site Description (Natural Stream)** | **SITE ID** | **General Collection Site Description (Natural Stream)** | **SITE ID** | **General Collection Site Description (Irrigation)** |
| --- | --- | --- | --- | --- | --- |
|  |  |  |  |  |  |
| **1** | End of natural stream near the rice field, with presence of human waste noted | **11** | Bank of natural stream, lush vegetation, taro (*C. esculenta*) garden | **12** | Area near the fishpond, adjacent to rice field |
| **2** | Bank of natural stream adjacent to rice field | **21** | Bank of natural stream with muddy surrounding and lush vegetation | **13** | Bank of irrigation canal adjacent to the rice field |
| 3 | Bank of natural stream adjacent to rice field and pig pen | 22 | Bank of natural stream with lush vegetation | 14 | Bank of irrigation canal adjacent to the rice field |
| **4** | Bank of natural stream adjacent to rice field, close to human habitation | **23** | Bank of natural stream with lush vegetation | **15** | Bank of irrigation canal adjacent to the rice field |
| **5** | Bank of natural stream, carabaos were observed | **24** | Bank of natural stream with bamboo bridge, lush vegetation, carabaos wallowing in the water | **16** | Bank of irrigation canal with water pump, carabao wallow |
| **6** | Bank of natural stream, carabaos were observed | **25** | Bank of natural stream with lush vegetation | **17** | Bank of irrigation canal adjacent to the rice field |
| **7** | Bank of natural stream, carabaos were observed | **26** | Bank of natural stream with lush vegetation | **18** | Bank of irrigation canal adjacent to the rice field |
| **8** | Bank of natural stream surrounded with lush vegetation | **27** | Bank of natural stream with lush vegetation, carabaos wallow in the water | **19** | Bank of irrigation canal adjacent to rice field |
| **9** | Bank of natural stream with water pump to rice paddy | **28** | Bank of natural stream with lush vegetation | **20** | Bank of irrigation canal adjacent to rice field |
| **10** | Bank of natural stream adjacent to rice field and taro (*Colocasia esculenta*) garden | **29** | Bank of natural stream with lush vegetation connecting to irrigation | **30** | Bank of irrigation canal surrounded with deep carabao wallow |
| Supplementary File 1. Characteristics of the 30 soil sampling sites based on ocular observations in Ekiran Village, Alangalang, Leyte, Philippines | | | | | |

**Supplementary File 1**
